# Essential Functional Modules for Pathogenic and Defensive Mechanisms in *Candida albicans* Infections

**DOI:** 10.1155/2014/136130

**Published:** 2014-03-18

**Authors:** Yu-Chao Wang, I-Chun Tsai, Che Lin, Wen-Ping Hsieh, Chung-Yu Lan, Yung-Jen Chuang, Bor-Sen Chen

**Affiliations:** ^1^Institute of Biomedical Informatics, National Yang-Ming University, Taipei 11221, Taiwan; ^2^Laboratory of Control and Systems Biology, Department of Electrical Engineering, National Tsing Hua University, Hsinchu 30013, Taiwan; ^3^Institute of Communications Engineering, National Tsing Hua University, Hsinchu 30013, Taiwan; ^4^Institute of Statistics, National Tsing Hua University, Hsinchu 30013, Taiwan; ^5^Department of Life Science and Institute of Molecular and Cellular Biology, National Tsing Hua University, Hsinchu 30013, Taiwan; ^6^Department of Medical Science and Institute of Bioinformatics and Structural Biology, National Tsing Hua University, Hsinchu 30013, Taiwan

## Abstract

The clinical and biological significance of the study of fungal pathogen *Candida albicans* (*C. albicans*) has markedly increased. However, the explicit pathogenic and invasive mechanisms of such host-pathogen interactions have not yet been fully elucidated. Therefore, the essential functional modules involved in *C. albicans*-zebrafish interactions were investigated in this study. Adopting a systems biology approach, the early-stage and late-stage protein-protein interaction (PPI) networks for both *C. albicans* and zebrafish were constructed. By comparing PPI networks at the early and late stages of the infection process, several critical functional modules were identified in both pathogenic and defensive mechanisms. Functional modules in *C. albicans*, like those involved in hyphal morphogenesis, ion and small molecule transport, protein secretion, and shifts in carbon utilization, were seen to play important roles in pathogen invasion and damage caused to host cells. Moreover, the functional modules in zebrafish, such as those involved in immune response, apoptosis mechanisms, ion transport, protein secretion, and hemostasis-related processes, were found to be significant as defensive mechanisms during *C. albicans* infection. The essential functional modules thus determined could provide insights into the molecular mechanisms of host-pathogen interactions during the infection process and thereby devise potential therapeutic strategies to treat *C. albicans* infection.

## 1. Introduction

In daily life, human beings are exposed to environments containing a wide variety of microorganisms. It is inevitable that humans will sometimes face opportunistic threats posed by some of these microbes. Pathogens, microorganisms that cause their host disease, have evolved numerous strategies to invade their hosts, while hosts have also evolved corresponding defensive responses to these invading agents [1]. The result of such host-pathogen interactions can result in damage to or even death of the host. Therefore, investigating the mechanisms of host-pathogen interactions may help biologists and clinicians better understand the underlying biological scenario. Once the pathogenic mechanisms of pathogens and the corresponding defensive mechanisms employed by hosts are uncovered, novel strategies that assist hosts in responding to microbial infection may be developed.

*Candida albicans*, a fungal pathogen, is a kind of ubiquitous commensal yeast that inhabits the mouth, gastrointestinal tract, and the vagina in humans. Under normal conditions,* C. albicans* is harmless to humans. However, it can induce serious mucosal and life-threatening systemic infections in individuals who are immunocompromised due to such factors as infection with human immunodeficiency virus (HIV), organ transplantation, or cancer chemotherapy. In addition,* C. albicans* is a major cause of hospital-acquired infection [2, 3].* C. albicans* has many morphological forms including a yeast form, a pseudohyphal form, and a hyphal form. The ability to switch from the yeast to hyphal form has been proposed as one of the major factors accounting for the virulence of the organism, and other studies have demonstrated that nonfilamentous* C. albicans* mutants are avirulent [4–6].

Until recently, the mouse, the fruit fly, and the wax moth were the main model organisms for studies of* C. albicans *infection. However, there are certain disadvantages in using these organisms in such models. The fruit fly and the wax moth lack adaptive immunity [7, 8], and the mouse is too expensive for large-scale experiments. Therefore, Chao et al. [9] developed the zebrafish (*Danio rerio*) model as a minivertebrate host system for* C. albicans *infection studies. They showed that* C. albicans* can invade zebrafish and kill the host in a dose-dependent manner [9]. Brothers et al. also developed the zebrafish larva as a transparent vertebrate model of disseminated candidiasis, showing that the infection model reproduces many aspects of candidemia in mammalian hosts [10]. In addition, the zebrafish undergoes rapid embryonic development and requires relatively small spaces in which to breed, leading to low experimental costs and making it a suitable infection model organism. Furthermore, the zebrafish has both innate and adaptive immune systems [11] and therefore has become widely used in the study of human diseases [12].

Several studies have identified the virulence factors and the corresponding virulence-associated genes in* C. albicans* [13]. Other studies have investigated immune responses occurring during the infection process, especially pathogen recognition mechanisms [14]. However, these studies have mainly focused on specific genes and their particular roles in the infection process and have not investigated host-pathogen interaction from a systems point of view [15]. In the light of experimental observations in which about 50% of zebrafish were seen to die of extensive bleeding 18 hours after being infected with* C. albicans* (1 × 10^8^ CFU) [9], we aimed to investigate both the functional modules of the activated pathogen essential in the invasion of zebrafish by* C. albicans* and the zebrafish functional modules likely to be responsible for defensive responses and the extensive bleeding. In other words, the goal of this study was to investigate the pathogenesis of* C. albicans* in fatal infections of zebrafish and the important defensive mechanisms employed by zebrafish against* C. albicans* infection from the network systems perspective. As a consequence, we simultaneously quantified the time-course gene expression profiles for both* C. albicans* and zebrafish during* C. albicans* infection. With the help of simultaneous host-pathogen interaction microarrays and other high-throughput omics data, the early-stage infection and late-stage infection protein interaction networks in both* C. albicans* and zebrafish were constructed. Protein-protein interactions are at the core of the intercellular interactions and control major biological functions. Differential interactions imply mechanistic changes that are a result of an organism's response to environmental conditions [16]. In the case of host-pathogen interaction, analyzing the differential interactions in a time-dependent manner can show how the host attempts to respond to the pathogen and how the pathogen responds within the host [16]. Therefore, changes in PPIs during infection may affect the pathogenesis of pathogens, while the reconfiguration of the protein-protein interactions in the host may reflect the activation of defensive mechanisms against pathogens. Using these constructed PPI networks, proteins with significant changes in their interaction profiles were considered to play important roles in infection pathology. Furthermore, from the identification of such significant proteins, the* C. albicans* functional modules associated with pathogenesis and the zebrafish functional modules involved in defense against* C. albicans* infection could be identified. It is hoped that by understanding the underlying pathogenic/defensive interaction mechanisms between the host and pathogen, biologists and clinicians may better appreciate how the pathogen infects its host and thereby devise effective therapeutic strategies to prevent loss of life in cases of* C. albicans *infection [17].

## 2. Materials and Methods

### 2.1. Omics Data Selection

In order to investigate important functional modules in* C. albicans*-zebrafish interactions, high-throughput omics data from many different sources were integrated, including simultaneous time-course gene expression profiles of* C. albicans* and zebrafish interactions obtained from microarray data, protein-protein interaction information from* Homo sapiens* and* Saccharomyces cerevisiae*, and ortholog data between humans and zebrafish and between* S. cerevisiae* and* C. albicans*. The time-course gene expression data were obtained from the GEO database (accession number: GSE32119). Experiments were performed to obtain* in vivo* genome-wide gene expression profiles simultaneously for both* C. albicans* and zebrafish during* C. albicans*-zebrafish interactions. Wild type AB strain zebrafish were intraperitoneally injected with* C. albicans* cell suspensions (SC5314 strain), and gene expression in both* C. albicans* and zebrafish was then assessed at nine subsequent times: 0.5, 1, 2, 4, 6, 8, 12, 16, and 18 hours after infection (hpi); this was performed three times in total [20].

Since there is little available in terms of protein interaction maps in either* C. albicans* or zebrafish, protein-protein interaction (PPI) information for these organisms was inferred from the interactome of* S. cerevisiae *and humans with the help of ortholog data [21]. Both the PPI data of* S. cerevisiae* and of humans were acquired from the Biological General Repository for Interaction Datasets (BioGRID) (http://thebiogrid.org/) [22]. The ortholog data pertaining to* C. albicans* and* S. cerevisiae* were retrieved from the* Candida *Genome Database (CGD) (http://www.candidagenome.org/) [23]; the ortholog data pertaining to zebrafish and humans were taken from the Zebrafish Model Organism Database (ZFIN) (http://zfin.org) [24] and the InParanoid database (http://InParanoid.sbc.su.se) [25]. In addition, gene annotations of* C. albicans* and zebrafish were obtained from CGD, the Gene Ontology (GO) database (http://www.geneontology.org/) [26], and the Database for Annotation, Visualization and Integrated Discovery (DAVID) (http://david.abcc.ncifcrf.gov/) [27].

### 2.2. Selection of Protein Pool

**Figure 1:**
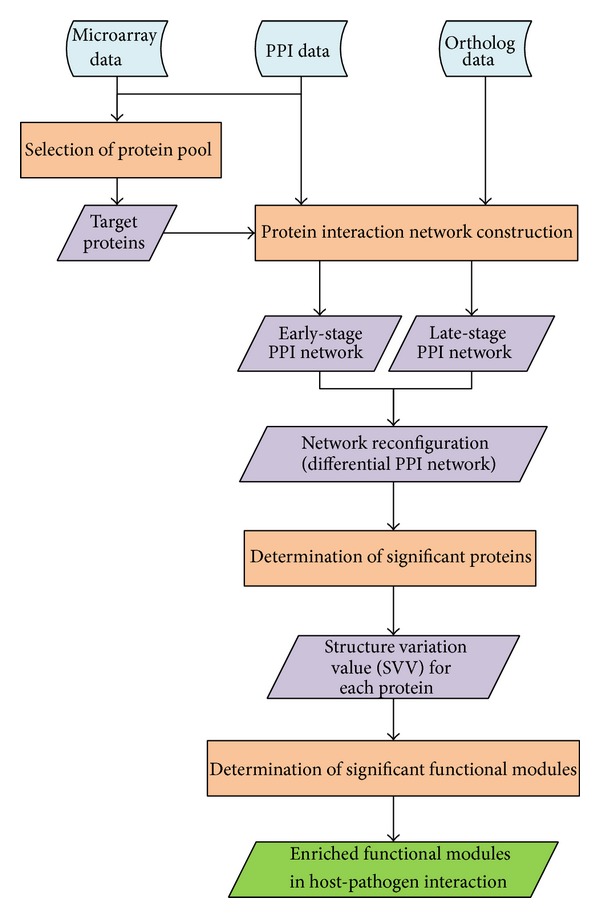
A flowchart for construction of protein-protein interaction networks and determination of enriched functional modules in host-pathogen interaction by comparing the early-stage and late-stage PPI networks. This figure shows the adopted approach in flowchart form. Blue boxes show the data sought in this study. Orange boxes indicate the steps used in the data-gathering process. Purple boxes represent the results of each processing step, and the green box denotes the final result of the whole approach.

The overall flowchart illustrating the approach adopted is shown in Figure 1. For both host data and pathogen data, gene expression profiles, ortholog data, and PPI data were used to construct dynamic PPI networks. In order to integrate gene expression profiles and PPI information, gene expression values were overlaid on the corresponding proteins as the protein expression levels [28]. Since the systems approach adopted in this study was based on dynamic protein-protein interaction networks, the extent of coverage of the interactome needed to be considered when selecting proteins of interest. For* C. albicans*, the PPI information was inferred from the interactions of* S. cerevisiae*, the best studied model system. However, the zebrafish has a much lower overall coverage in terms of protein interaction maps than* C. albicans*. Therefore, the selection of the protein pool was different for* C. albicans* and zebrafish. One-way analysis of variance (ANOVA) was employed to select differentially expressed proteins in* C. albican*s. The null hypothesis of ANOVA assumed that the average expression level of a protein would be the same at every time point [29]. Proteins with Bonferroni adjusted *P* values of less than 0.1 were selected in the protein pool as target proteins. For zebrafish, the protein pool included all proteins even though they were not differentially expressed. Since PPI networks were used in this study, those target proteins for which PPI information was not available were filtered out of the protein pool.

### 2.3. Protein Interaction Network Construction

Our strategy was to identify proteins significant in protein interaction network reconfiguration during the infection process and then to investigate the enriched functional modules composed of these significant proteins. For this reason, early- and late-stage PPI networks for both* C. albicans* and zebrafish were constructed for network configuration comparison. Previous histological analysis showed that the first zebrafish was seen to die 5 hours after being infected with* C. albicans* (1 × 10^8^ CFU) and about 50% of zebrafish had died by 18 hpi [9]. Therefore, the gene expression data taken nine time points after infection were separated into two groups; one contained the 0.5–4 hpi data, the early stage of infection, and the other comprised of the 4–18 hpi data, the late stage of infection. Therefore, the PPI networks constructed from gene expression data within the 0.5–4 hpi period were designated as the early- stage PPI networks, and the gene expression data after 4 hpi were used to construct the late-stage PPI networks. With data pertaining to the proteins in the protein pool and the protein-protein interaction data obtained from the database mining, a rough PPI network for both post-infection stages was constructed for* C. albicans* and zebrafish by linking the proteins with the PPI information. However, under the specific conditions of the infection process, these rough PPI networks may be inappropriate because they were constructed from data obtained under all possible experimental conditions in the literature and databases. Therefore these rough PPI networks needed refining into a suitable network occurring specifically during infection with the help of gene expression profiles. In this study, a discrete dynamic model was employed to determine the PPI networks that occurred in the infection of zebrafish by* C. albicans* [30] (see Supplementary methods for details available online at http://dx.doi.org/10.1155/2014/136130). Based on the time-course microarray data, the system parameter estimation method and the model selection measurement Akaike Information Criterion (AIC) were then used to detect significant interactions [31, 32] (see Supplementary methods for details). In this way, with different sets of microarray data (0.5–4 hpi for the early stage and 4–18 hpi for the late stage), two refined PPI networks were constructed for the early and late stages of* C. albicans* infection of zebrafish for both organisms. These early and late stage PPI networks will be compared with each other to ascertain their network reconfigurations in the infection process.

### 2.4. Network Reconfiguration between the Early and Late Stages of the Infection Process

Living organisms take appropriate actions to respond to diverse environmental changes and internal perturbations. Through adjustment of their molecular interactions, organisms tend to maintain a proper, beneficial, or stable state in response to changes in such conditions [33]. Therefore the protein-protein network changes with these interaction variations over time to balance out the effects of environmental changes; that is, the PPI network reconfigures as corresponding protein interactions change to respond to the different conditions brought about by the infection process. A matrix indicating significant protein-protein interactions in the refined PPI network was constructed from those identified protein interaction abilities (see Supplementary methods). The established PPI interaction matrix of a refined PPI network can be thus represented:

(1) $$\begin{array}{c} \left[\begin{array}{cccc} b_{11} & b_{12} & \ldots & b_{1K}\\ b_{21} & b_{22} & \ldots & b_{2K}\\ \vdots & \vdots & \ddots & \vdots \\ b_{K1} & b_{K2} & \ldots & b_{KK} \end{array}\right], \end{array}$$

where *b*_*ij*_ denotes the identified interaction ability between proteins *i* and *j* and *K* represents the number of proteins in the refined PPI network. Hence, the interaction matrix of the differential PPI network contrasting the early-stage with late-stage PPI networks was expressed as follows:

(2) Dl=[d11,ld12,l…d1K,ld21,ld22,l…d2K,l⋮⋮⋱⋮dK1,ldK2,l…dKK,l]=[b11,2l−b11,1lb12,2l−b12,1l…b1K,2l−b1K,1lb21,2l−b21,1lb22,2l−b22,1l…b2K,2l−b2K,1l⋮⋮⋱⋮bK1,2l−bK1,1lbK2,2l−bK2,1l…bKK,2l−bKK,1l],

where *d*_*ij*,*l*_ indicates the change of protein interaction ability of the *l*th organism system between late-stage PPI network and early-stage PPI network for protein *i* and protein *j*, *b*_*ij*,1*l*_ and *b*_*ij*,2*l*_ represent the identified protein interaction ability between protein *i* and protein *j* for the early-stage PPI network and the late-stage PPI network of the *l*th organism, respectively, and *l* denotes the pathogen or host. Therefore, for each organism system, a matrix *D*_*l*_ was established to show the differential PPI network between the early-stage and late-stage PPI networks. Furthermore, the structural variations between the early-stage and late-stage PPI networks can be determined by the differential PPI network for each protein. The structure variation value (SVV) is considered to be an index to quantify the PPI network reconfiguration between these two stages in the infection process:

(3) $$\begin{array}{c} {\text{SVV}}_{l}=\left[\begin{array}{c} {\text{SVV}}_{1,l}\\ {\text{SVV}}_{2,l}\\ \vdots \\ {\text{SVV}}_{K,l} \end{array}\right], \end{array}$$

where SVV_*i*,*l*_ = ∑_*j*=1_^*K*^|*d*_*ij*,*l*_|, *l* = host or pathogen, and *i* = 1,…, *K*; that is, the reconfiguration of the protein *i* of the *l*th organism is obtained from the absolute sum of the *i*th row of *D*_*l*_ in (2) and the reconfiguration of the PPI network is obtained by the vector SVV_*l*_ for the *l*th organism. For a protein *i* of the *l*th organism system, SVV_*i*,*l*_ implies the extent of the structure change of the *i*th protein between the early stage and late stage of the infection process. SVV_*l*_ denotes the network reconfiguration of the *l*th organism.

### 2.5. Investigation of Significant Functional Modules in the Infection Process

During the host-pathogen interaction, the pathogen makes modifications to its PPI network for invasive purposes, while the host makes adjustments to its protein levels to defend itself against the pathogen. The participation of a protein in a specific biological process is correlated with the changes it undergoes in the PPI during that process. In this study, changes in the PPI structures in both organisms (i.e., the differential PPI networks) between the early and late stages of infection revealed proteins with significant SVVs, which were then considered to play important roles in the infection process. Furthermore, functional modules made up of proteins with significant SVVs were regarded as important factors in the specific biological behavior of the infection process. Since no zebrafish died in the early stage (0.5–4 hpi) and the infected fish started to die in the late stage (4–18 hpi), these significant functional modules were considered to be important in conferring the virulence of the pathogen for* C. albicans*. In the case of zebrafish, these significant functional modules were possibly associated with defensive mechanisms by which certain biological processes were activated or inhibited in order to respond to* C. albicans* infection.

In order to determine the significance of the SVV of a given protein, an empirical *P* value was computed. A null distribution of SVVs was created based on the SVVs of random PPI networks. The random PPI networks were generated by permuting the network structure with the network size being constrained; that is, Erdős-Rényi random graph model was used to create the random PPI networks with the same number of protein interactions. Hence, the SVVs for each protein in the random PPI networks could be computed. With 100,000 iterations, the *P* value of a given SVV was calculated as the fraction of the number of random PPI networks in which that SVV was at least as large as the SVV of the real PPI network. SVVs with *P* values ≤ 0.05 were considered to be significant and the corresponding proteins were assumed to undergo significant changes in their interaction characteristics during the infection process.

Once proteins with significant SVVs were thus found in* C. albicans *interactions with zebrafish, gene annotations from the GO and DAVID databases were used to form enriched functional modules composed of the proteins identified as having significant SVVs. As time and environment change, the activated functional modules in an organism also change. We believe that the functional modules composed of proteins with significantly elevated SVVs in* C. albicans *are responsible for its pathogenesis. Likewise, enriched functional modules in infected zebrafish might be the major functional modules for defensive response induced by* C. albicans* infection.

## 3. Results 

### 3.1. Construction of Dynamic PPI Networks and Identification of Significant Proteins in* C. albicans* Infection

Using the methods outlined above, several functional modules tentatively accounting for* C. albicans* pathogenicity and the associated defensive responses of zebrafish were investigated for their possible roles in the infection process. For* C. albicans*, the protein-protein interaction network was made up of 1,369 differentially expressed proteins selected in the protein pool. For zebrafish, a total of 7,861 proteins were all selected in the protein pool for PPI network construction without considering whether they were differentially expressed.

Although a rough PPI network can be constructed from target proteins along with PPI information obtained from database mining, it was more appropriate to refine the rough PPI network by microarray data of* C. albicans* infections. In this way, the refined early-stage and late-stage PPI networks were established for both* C. albicans* and zebrafish. For* C. albicans*, the early-stage PPI network was constructed from 1,318 proteins and 2,902 PPIs; the late-stage PPI network was comprised of 1,301 proteins and 4,045 PPIs. For zebrafish, there were 13,399 PPIs and 6,689 proteins in the early-stage PPI network and 18,807 interactions among 7,023 proteins in the late-stage PPI network. The extent of which a protein was considered significant in the infection process was based on the changes in the PPIs, that is, in the differential PPI networks (Supplementary Figure S1 and Figure S2 for* C. albicans* and zebrafish, resp.). In other words, significant proteins were identified by the comparison between edge variations of the PPI networks in the two stages of the infection process, as revealed by the SVVs in the differential PPI networks with *P* values ≤ 0.05 (distributions of SVVs for* C. albicans* and zebrafish were shown in Supplementary Figure S3). In this way, 139* C. albicans* proteins were found to be of significance during the course of infection, and 380 zebrafish proteins were identified as playing important roles in defensive processes against* C. albicans*. Some functionally enriched modules comprised of these SVV-significant proteins are discussed in the following sections.

### 3.2. Investigation of Essential Functional Modules for Pathogenic and Defensive Mechanisms in* C. albicans* Infection

#### 3.2.1. Functionally Enriched* C. albicans* Modules for Pathogenic Mechanism in the Infection Process

**Figure 2:**
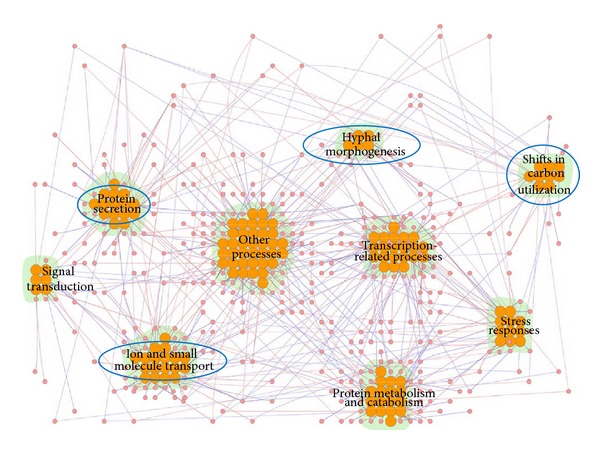
The functional modules composed of 139* C. albicans* proteins found to be significant for pathogenic mechanisms in the infection process. This figure shows the differential PPI network constructed from 139* C. albicans* proteins significant in the infection process and the interactions among them. Red and blue edges indicate positive and negative *d*_*ij*,*l*_ values, respectively, as calculated using (2). The orange nodes represent the significant proteins, that is, proteins with SVV *P* values ≤ 0.05. There were nine enriched* C. albicans* functional modules occurring in host-pathogen interactions, playing roles in such processes as hyphal morphogenesis, ion and small molecule transport, protein secretion, shifts in carbon utilization, stress responses, protein metabolism and catabolism, signal transduction, transcription-related processes, and other processes. The functional modules marked with blue circles were investigated in this study. The figure was created using Cytoscape plugin Cerebral [18, 19]. The names of the proteins have been omitted for simplicity.

The 139 SVV-significant proteins seen in* C. albicans* can be divided into nine functional modules using annotations from the GO database. The nine modules are associated with hyphal morphogenesis, ion and small molecule transport, protein secretion, shifts in carbon utilization, stress responses, protein metabolism and catabolism, signal transduction, transcription-related processes, and other processes (proteins not belonging to the above-mentioned functional modules or lacking GO annotation, Figure 2). Eight out of nine functional modules are statistically enriched beyond what is expected by chance (*P* < 0.05, Fisher's exact test, except for other processes). Some of these nine functional modules are associated with general biological processes. Therefore, in this study, we have focused only on four of these modules: those playing a role in hyphal morphogenesis, ion and small molecule transport, protein secretion, and shifts in carbon utilization (Figure 2). We infer that these four enriched functional modules account for pathogenesis of* C. albicans* in infecting zebrafish, and further discussion of them is given below (Table 1).

**Figure 3:**
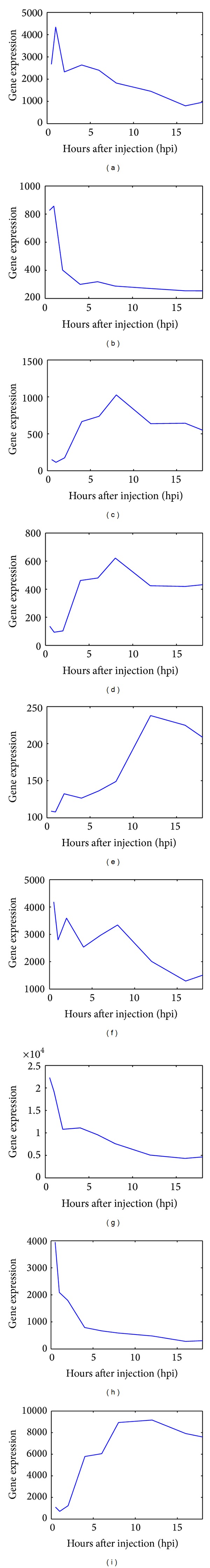
Gene expression profiles of* C. albicans* proteins in the functional module underlying shifts in carbon utilization: (a) Lat1, (b) orf19.3782, (c) Tes15, (d) orf19.4121, (e) Tes1, (f) Pyc2, (g) Acc1, (h) Agp2, and (i) Pox1-3.

(1) Hyphal morphogenesis: the fungal pathogen* C. albicans* grows in various morphogenic forms. The pathogen can exist in yeast form or undergo a process of morphogenesis to develop pseudohyphae or polarized hyphae. It has been demonstrated that the morphogenetic plasticity of* C. albicans* correlates closely with its pathogenicity and the phenotypic switch of the yeast to its hyphal form is a crucial factor in* C. albicans* pathogenesis [6]. Generally, mutant strains of* C. albicans* defective in the ability to form hyphae are less virulent in animal models [5, 34]. From a systematic level, considering the change of network structure between the two infection stages, several proteins with significant SVVs were included in the functional module, such as Cas4 and Als3 (Table 1).* PAG1*, alternatively known as* CAS4*, is a gene in the RAM network, a conserved signaling network that regulates polarized morphogenesis. The* CAS4* mutant showed hypersensitivity to cell wall-perturbing agents and loss of cell polarity [35]. Additionally, the hyphal form can interact with the yeast and pseudohyphal forms to produce biofilms, which can act as a source of recurrent infection and play an important role in resistance to antifungal agents [36]. Als3 is involved in biofilm formation and an* ALS3* mutant has been shown to be biofilm-defective* in vitro* [37]. In short, during* C. albicans* infection, morphogenesis was a crucial virulence-determining factor observed in the network structure variations between the early and late stages of infection.

(2) Ion and small molecule transport:* C. albicans* can thrive in various niches within its host, such as mouth, gastrointestinal tract, and the vagina. These niches are characterized by their diverse environments, with extreme variation in pH and nutrient composition. As a consequence,* C. albicans *must either adapt or, more likely, alter its niche in order to survive, possibly resulting in damage to the host tissue. Several proteins with significant SVVs were included in this functional module, such as Tna1, Agp2, and Can1, proteins responsible for nicotinic acid, carnitine, and amino acid transport, respectively (Table 1). These transporters, all showing significant interaction variations in network structures between the early and late stages of* C. albicans* infection, seemed to indicate that a reorganization of substrate uptake and utilization occurs. This reorganization could also enable* C. albicans* to assimilate available nutrients from its host allowing it to survive in a hostile microenvironment. Furthermore, using GO annotations, proteins like Als3, Mac1, and orf19.3769, which are involved in the transport of such metal ions as iron, copper, and zinc, were also identified. Copper, zinc, and iron are all examples of nutritionally essential trace elements, also referred to as micronutrients. These minerals are required for growth and the optimal function of many organisms. Both excess and deficiency will have adverse effects in such organisms. Thus maintaining an adequate supply of micronutrients is critical for the viability of* C. albicans*. Previous research has shown that calprotectin from the cytoplasm of neutrophils can inhibit* C. albicans* growth through competition for zinc [38]. Therefore adequate zinc levels are required for* C. albicans* growth. In addition, superoxide dismutases cofactored with copper and zinc (Cu/ZnSOD) are found in* C. albicans* [39]. These enzymes play important roles in antioxidant defense when cells are exposed to oxygen. Antioxidants can inhibit oxidation reactions, which can produce free radicals, leading to cell damage or death. A previous study demonstrated that* C. albicans* lacking Cu/ZnSOD was more susceptible to macrophages and its virulence was seen to attenuate in mice [40]. Hence it would seem that the uptake of copper and zinc appears to be crucial in* C. albicans* growth and progression of infection. It is well known that* C. albicans* possesses iron acquisition mechanisms that are recognized to be essential for hyphal growth in the infection process [41]. Such mechanisms could also deprive the host of iron thereby exerting a harmful effect. Als3 is a hyphal-associated adhesin and invasin in* C. albicans* and is essential in ferritin-binding to the external hyphal layer [42]. Ferritin is an iron-containing host protein and therefore a potential iron source for pathogens. Previous studies have indicated that* C. albicans* mutants lacking* ALS3* displayed defective ferritin-binding abilities and that this also attenuated the pathogenic damage done to oral epithelial cells [41]. This distinctive iron-utilization characteristic contributes to the survival of the organism and pathogenesis in the host and also seems to play a role in hyphal formation, adhesion, and invasion during host-pathogen interactions. Taken together, ion and small molecule uptake and utilization enable the pathogen to adapt, invade, or even damage the host in* C. albicans* infection.

(3) Protein secretion: in the light of data from the differential PPI networks between the early and late infection stages, proteins having a role in protein secretion, such as Sap4 to Sap6, Sro77, and Sys3, showed significant network structure variations (Table 1). It is known that every cell is contained within a membrane that separates its interior from the external environment. Hence, the protein secretion system, which transports or extrudes molecules from the interior of microbes to the external environment, is an important mechanism by which microbes adapt and survive in their environments [43]. Several adhesins and extracellularly secreted hydrolases, such as secreted aspartyl proteinases (SAPs), secreted lipases (LIPs), and phospholipase B (PLB) [44], were identified as contributing to the virulence of* C. albicans*, and it is known that these proteins facilitate nutrient supplies, adhesion to host cells, tissue invasion, and even host cell damage. Dynamic changes to cell surface components and proteins released from the pathogen into the host cell environment can play important roles in host-pathogen interactions. That is to say, the virulence characteristics of* C. albicans* are closely related to its protein secretion mechanisms during infection. The Golgi apparatus is known to be involved in the secretion mechanism. Studies have demonstrated that in* C. albicans*, the Golgi complex, consisting of puncta, is redistributed to the distal portion of the extending hyphae whereas it is randomly distributed throughout the cytoplasm in the yeast form [45]. Since the Golgi apparatus is considered a locus of biomolecule manufacture, the relocation of the Golgi to the distal hyphal tip during hyphal formation means that post-Golgi secretory vesicles do not need to undergo long-distance transport from the cell body to the growing apical tip. Therefore, such Golgi redistribution would provide more rapid apical growth and result in further efficiency of the infection process [43, 45]. It also seems that clustered Golgi puncta give* C. albicans* the ability to secrete virulence-related proteins that adhere to, invade, or damage host tissue during host-pathogen interactions. However, there is no explicit evidence, except the well-known example of Sap4 to Sap6 [46], showing that mutant strains deficient in these identified protein secretion-related genes exhibit reduced virulence. Additional studies are needed to fully investigate the relationship between protein secretion and pathogenesis. Despite this, protein secretion mechanisms certainly enable* C. albicans* to adapt, survive, and invade the host, and therefore play an important role in host-pathogen interaction.

(4) Shifts in carbon utilization: once host immune cells recognize and attach to the pathogen-associated molecular patterns (PAMPs) of pathogens, phagocytosis is activated, in which pathogens are engulfed by the cell membranes of phagocytes to form an internal phagosome. Phagocytosis is a major cellular process used by hosts to destroy and remove pathogens. Some studies have shown that the phagosome of the host is a nutrient-poor environment for pathogens [47]. Moreover,* C. albicans* has been shown to undergo carbon starvation and glucose deprivation after internalization by macrophages [47]. Although glucose generally serves as the preferred source of energy and precursor for the synthesis of several other substances, the glucose-deficient environment of the macrophage requires carbon metabolism by the pathogen to be modulated after phagocytosis. Previous studies have shown that genes responsible for controlling the glyoxylate cycle and gluconeogenesis, which are used for the assimilation of two carbon compounds, are activated after* C. albicans* is exposed to macrophages. Additionally, acetyl-CoA is a precursor which can drive the glyoxylate cycle or gluconeogenesis and which could be derived from fatty acids from either* C. albicans* or the macrophage [47]. In this study, by looking at proteins with significant SVVs, a functional module of carbon utilization-related genes including Lat1, orf19.3782, and Tes15 was determined (Table 1). Annotation data from the* Candida *Genome Database revealed that Lat1 and orf19.3782 are associated with acetyl-CoA biosynthesis or transport. Tes15, orf19.4121, and Tes1 are involved in acyl-CoA metabolic processes; acyl-CoA is a coenzyme involved in the metabolism of fatty acids. Pyc2 is involved in the process of gluconeogenesis. Acc1, Agp2, and Pox1-3 are involved in fatty acid biosynthesis, metabolic processes, and oxidation. It is speculated that during the early stage of infection,* C. albicans* is internalized by macrophages and some acetyl-CoA-, acyl-CoA-, and fatty acid-associated proteins, such as Lat1, Tes15, and Agp2 are activated (Figure 3). Nevertheless, during the late-stage of* C. albicans *infection, macrophages are killed and* C. albicans* begins to disseminate. When this occurs, glucose is used as the chief carbon source, causing the acetyl-CoA-associated and fatty acid-associated proteins to downregulate (Figure 3). However, the trends in the gene expression profiles of Tes15, orf19.4121, Tes1, and Pox1-3 are different from the other five proteins (Figure 3). This gradually increasing and subsequently decreasing expression profile suggests the induction of acetyl-CoA synthesis during infection. A possible explanation is that there is still some* C. albicans* that have been phagocytosed and therefore fatty acid-associated proteins are needed to produce glucose. Taken together, the rapid adaptation to ever-changing host environments, such as the reorganization of carbon utilization, enables* C. albicans* to survive and infect the host.

#### 3.2.2. Functionally Enriched Zebrafish Modules for Defensive Mechanism during* C. albicans* Infection

**Figure 4:**
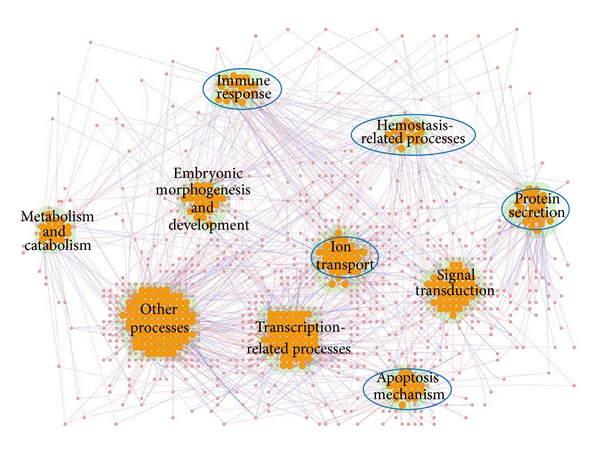
The functional modules composed of 380 zebrafish proteins found to be significant for defensive mechanisms in the infection process. This figure shows a differential PPI network constructed from 380 infection-significant zebrafish proteins and their interactions. Red and blue edges indicate positive and negative *d*_*ij*,*l*_ values, respectively, calculated using (2). The orange nodes represent significant proteins, that is, proteins with SVV *P* values ≤ 0.05. There were ten enriched zebrafish functional modules occurring in host-pathogen interactions, including those underlying immune response, apoptosis mechanism, ion transport, protein secretion, hemostasis-related processes, signal transduction, transcription-related processes, embryonic morphogenesis and development, metabolism and catabolism, and other processes. The functional modules marked with blue circles were investigated in this study. The figure was created using Cytoscape plugin Cerebral [18, 19]. The protein names have been omitted for simplicity.

To investigate what functional modules of zebrafish were induced by* C. albicans* infection, the bioinformatics database DAVID [27] and GO annotations were used for the analysis of zebrafish proteins. By applying functional annotation clustering in DAVID, 380 proteins identified as having significant SVVs could be classified into ten functional modules. These functional modules represent immune response, apoptosis mechanism, ion transport, protein secretion, hemostasis-related processes, signal transduction, transcription-related processes, embryonic morphogenesis and development, metabolism and catabolism, and other processes (proteins not belonging to the above-mentioned functional modules or without GO annotation, Figure 4). Among these ten modules, nine are statistically enriched (*P* < 0.05, Fisher's exact test, except for other processes). Since some functional modules represent general biological processes, in this study we have only focused on five functional modules: immune response, apoptosis mechanism, ion transport, protein secretion, and hemostasis-related process (Figure 4). The first four functional modules are considered to play defensive roles in the battle of host against pathogen. The functional module of the hemostasis-related process can be used to explain the pathological outcome, that is, the fatal bleeding of zebrafish seen in the* C. albicans*-zebrafish infection model in this study. These defensive functional modules are now further discussed (Table 2).

**Figure 5:**
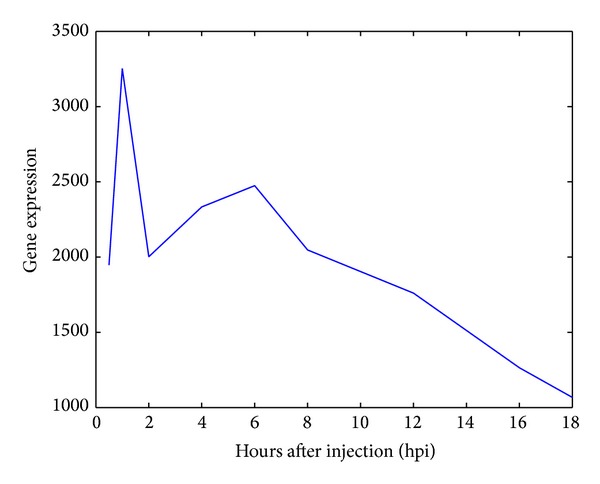
The gene expression profile of zebrafish laminin, alpha 4 (Lama 4).

(1) Immune response: it is well known that* C. albicans* can be both a harmless commensal organism and a fatal pathogen. The transition from commensal organism to pathogen is dependent on the interaction between* C. albicans* and the host innate immune system. Several proteins with significant SVVs identified from the network structure variations during infection are involved in the immune response, such as Tlr2 and B2m (Table 2). During the interaction between host and pathogen, when the pattern recognition receptors (PRRs) of the host cells recognize fungal pathogen-associated molecular patterns (PAMPs), an innate response is usually triggered to combat the pathogen. These PRRs include various toll-like receptors (TLRs) which are expressed by different cell types, such as macrophages, monocytes, and dendritic cells, and are the primary immune sensors for the detection of invading pathogens [48]. One of the mechanisms involved in the innate immune recognitions of fungal pathogens is mediated by the Dectin-1/Tlr2 receptor complex that recognizes *β*-glucan, a major component of the cell wall of* C. albicans* [49]. In addition, the activation of macrophages and dendritic cells expressing TLRs will also initiate the adaptive immune response. Beta-2 microglobulin (B2m), a protein found to be with significant SVV in this study, is associated with MHC (major histocompatibility complex) class I molecules, which helps T cells to recognize antigens. It has been shown that B2m-knockout mice do not express MHC class I molecules and they lack CD8+ and natural killer T cells [50]. If dysfunction of the host immune system were to occur, pathogens would invade easily resulting in severe damage or even life-threatening systemic infection. The identification of the enrichment of the functional module underlying the immune response reinforces the important role the immune system plays in defensive mechanisms against invasive* C. albicans* in the encounter between* C. albicans* and zebrafish in the infection process.

(2) Apoptosis mechanism: apoptosis is a biological process which results in cell death and the proper modulation of apoptosis is essential for the survival of the host. However, hosts and pathogens induce apoptosis in different ways so as to gain optimal benefit. Pathogens have evolved diverse strategies to induce or inhibit host cell apoptosis, allowing the pathogen to evade the innate response and favoring further dissemination of the pathogen into the host tissues [51, 52]. In contrast to pathogens, hosts defend against infection by inducing apoptosis in infected cells and inhibiting apoptosis in immune cells [53, 54]. It has been demonstrated that the outer surface of the cell wall of* C. albicans* is coated with phospholipomannan (PLM) and that PLM binds to the membranes of macrophages and stimulates Tlr2-mediated apoptosis [55]. This results in macrophages apoptosis and enhanced survival of* C. albicans* [55, 56]. In contrast, studies have shown that resistance of monocytes to* C. albicans*-induced apoptosis may limit pathogen replication, protect monocyte viability, and therefore enhance the host defense response [53, 57]. In addition, although there is no explicit evidence indicating direct apoptotic effects on the nonphagocytic cells of the host in* C. albicans* infection, it has been shown that apoptosis affords the infected intestinal epithelial cells a mechanism for defense against invasive enteric pathogens by destroying infected or damaged epithelia [58]. Thus, we suggest that zebrafish induces apoptosis in* C. albicans*-infected cells to prevent pathogen dissemination or to kill pathogens. Taken together, during the* C. albicans*-zebrafish interaction, it seems that* C. albicans* interferes with the apoptosis mechanism of zebrafish to elude host defense and infect host cells, while zebrafish manipulate apoptosis to help eliminate the threat posed by* C. albicans*. No evidence exists to indicate that mutants with these identified apoptosis-related genes knocked out have higher mortality rates or are more highly susceptible to pathogen infection, but it is reasonable to speculate that dysfunctions in zebrafish apoptosis mechanisms would weaken the ability of the fish to defend against* C. albicans*, and zebrafish lacking such genes would be infected more severely and eventually die. Thus, the enriched functional module pertaining to apoptosis plays an important role in the complex host-pathogen interaction.

(3) Ion transport: from observations of significant SVVs, several proteins involved in calcium, potassium, iron, and zinc ion transport have been identified as significant in infection (Table 2). Previous studies have shown that the regulation of Ca^2+^ and K^+^-signaling pathways is involved in T lymphocyte activation [59]. Additionally, intracellular calcium and potassium ion homeostasis influences apoptosis [60]. Therefore, proper calcium and potassium ion transport assists immune responses and apoptosis in zebrafish in response to* C. albicans* infection. In addition to calcium and potassium ions, there are also transporters that participate in micronutrient transport, such as iron and zinc. Micronutrients are nutrients required in small quantities to support normal physiological function. Pathogens have developed certain strategies to deprive the host of these micronutrients in order to promote growth and pathogenesis. Conversely, hosts sequester micronutrients from invading pathogens, that is, making these micronutrients unavailable to the pathogens, a concept termed nutritional immunity. Iron is an essential cofactor for several proteins and enzymes and, therefore, involved in numerous cellular functions and metabolic pathways [50]. A well-studied form of nutritional immunity is the iron-withholding defense system [61]. Using the approach adopted throughout this study, it was shown that transferrin-a (Tfa), a protein related to iron transport, undergoes a significant protein interaction change. Hosts have several iron-withholding mechanisms, and one of them acts through the host iron-binding proteins, the transferrins [41]. Thus, transferrin, responsible for iron scavenging in plasma and lymph, has antimicrobial activity in the host-pathogen interaction. Recent work has shown that there is also competition for micronutrients other than iron (zinc, e.g.) during the host-pathogen interaction [62]. Zinc, also essential for living organisms, plays a crucial role in the immune system, and zinc deficiency induces broad-spectrum defects in both innate and adaptive immunity [63]. Zinc sequestration by the host might inhibit microbial growth and protect against infection. A previous study has shown that calprotectin, a neutrophil-derived protein, competes with* C. albicans* for zinc which is needed for growth [64]. Taken together, it can be seen from the ion transport functional module investigated that it is reasonable to infer that normal ion transport systems, which limit micronutrient availability and are required for optimal immune or apoptotic function, assist zebrafish in defending against* C. albicans* infection.

(4) Protein secretion: like the situation in* C. albicans*, protein secretion or protein transport was identified as the enriched functional module during infection in zebrafish. Several proteins, especially those belonging to Rab family, showed significant network structure variations (Table 2). The Rab family is part of the Ras superfamily of small GTPases and functions in the regulation of intracellular vesicle trafficking and protein transport between different organelles and various secretory vesicles [65, 66]. Although there is no direct evidence linking zebrafish Rab family proteins with fungal infections, Rab GTPases have been found to be involved in the process of pathogen infection in many other organisms. In* C. elegans*, the small GTPase Rab1 was shown to control innate immunity by mediating antimicrobial peptide gene expression [67]. In red drum fish (*S. ocellatus*), it has recently been reported that Rab1 regulates intracellular bacterial infection and thus is likely to play a role in bacteria-induced host immune defense [66]. In mammals, Rab5 and Rab7 have been shown to govern the early events of HIV-1 infection in human placental cells [68]. Additionally, Rab5 and Rab7 were demonstrated to affect the entry and transport of some viruses and bacteria [66]. These studies have indicated that Rab proteins are functionally associated with endocytosis and trafficking of intracellular pathogens, and pathogens evolve corresponding strategies to modulate Rab functions [69]. Based on the findings from other organisms and the fact that many Rab proteins were identified as SVV-significance in this study, we infer that Rab proteins in zebrafish also play important roles in host resistance against microbial infections. Further studies are needed to characterize the functions of zebrafish Rab proteins, which would help understand the relationship between protein secretion and host response during infection.

(5) Hemostasis-related processes: in this study, the pathological outcome of the host-pathogen interaction in zebrafish was found to be massive fatal bleeding. Based on the pathological outcome and the fact that* C. albicans* may cause deep-seated infection and disruption of endothelial surfaces [70], it seems that damage of host endothelial cells or blood vessels might occur during the interaction of* C. albicans* with zebrafish. Conversely, from the molecular perspective, several proteins important in hemostasis-related processes were found to have significant SVVs (Table 2). The zebrafish protein Calcrla, previously named Crlr, and protein Lama4 belong to this functional module. The calcitonin receptor-like receptor (Crlr) is a main endothelial cell receptor involved in cardiovascular homeostasis. In zebrafish, it has been demonstrated that mutation of* crlr*, which is associated with vascular development and angiogenesis, leads to atrophy of the trunk dorsal aorta or lack of blood circulation [71]. In this study, from the decreased Calcrla edge numbers in the protein interaction network dynamics, we may infer that Calcrla-employing biological processes were attenuated in the late stage of infection. Blood vessels are composed of two major cell types: endothelial cells and periendothelial cells. In addition to these cellular components, there are certain structural elements involved in the preservation of vascular integrity, such as adherens junctions, basement membranes, and the extracellular matrix [72]. Laminins are components of the basement membrane. Zebrafish with morpholino knockdown of* lama4* have been demonstrated to undergo cardiac dysfunction and embryonic hemorrhage [73]. In this study, the gene expression of the identified zebrafish laminin, alpha 4 (Lama4), declined as infection advanced (Figure 5), indicating that vascular integrity may not be maintained. Therefore, from the behavior of the hemostasis-related functional module identified, we speculate that* C. albicans* can penetrate endothelial cells and invade deeper tissues in zebrafish. In addition, the blood vessels of zebrafish were found to be damaged and vascular homeostasis could not be maintained during the late stage of* C. albicans* infection.

## 4. Discussion 

The importance of host-pathogen interactions has long been apparent to biologists and clinicians. It is vital to understand the possible factors determining the virulence of pathogens during an infection process. Simultaneously, the host defensive mechanisms and pathogenic mechanisms of damage, disease, and even mortality of the host are also of interest to researchers. Once the underlying molecular mechanisms are unveiled, it should become possible to develop various therapeutic strategies to prevent tissue damage and death caused by* C. albicans* infection. In this study, the pathogenic functional modules of* C. albicans *active during infection and the corresponding defensive functional modules of zebrafish occurring in response to the pathogenic threat were investigated by simultaneous host-pathogen interaction microarray data from both the systematic and molecular viewpoints. Through gene expression profiles, protein-protein interaction information obtained from database mining, and discrete dynamic interaction models, PPI networks of pathogen and host were constructed at two different infection stages. By comparing the refined PPI networks at the early and late infection stages to generate a differential PPI network, the PPI network reconfiguration and those proteins showing significant interaction changes during the infection period were determined. Furthermore, enriched functional modules among those proteins identified as playing significant roles were investigated in great detail by GO annotation. Hyphal morphogenesis, ion and small molecule transport, protein secretion, and shifts in carbon utilization were found to be the most important molecular mechanisms of pathogenesis in* C. albicans *infection. At the same time, immune responses, apoptosis, ion transport, and protein secretion were found to be crucial molecular defensive mechanisms occurring in zebrafish in response to the pathogen. Additionally, we speculate from the functional module of hemostasis that* C. albicans* can damage the blood vessels of zebrafish, resulting in irreparable vascular destruction, which is consistent with the pathological outcome of fatal hemorrhage.

Biological systems are highly dynamic entities that continuously respond to environmental changes. However, few studies have investigated network reconfiguration or network rewiring to elucidate cellular responses [16]. The method employed in this study has been shown to be useful in constructing PPI networks and identifying the essential functional modules for pathogenic and defensive mechanisms in an infection process based on differential network analysis. Such an approach could highlight those interactions that changed dramatically across different conditions and potentially be suitable for the study of network comparisons with different cellular responses. Nevertheless, there are still some drawbacks to be addressed. First, the protein-protein interaction data for* C. albicans* and zebrafish used in this study were inferred from the interactomes of* S. cerevisiae *and humans with the help of corresponding ortholog data. Even though the imprecision in PPI information could lead to deviations between the constructed PPI network and the real situation, AIC was used to detect significant interactions under the specific condition of infection process with the help of gene expression profiles. In other words, the potential false positive PPIs that arose from ortholog-based inference could be pruned by AIC. In this case, the effect of the imprecise PPI information would be minimized. However, high coverage and reliable protein interaction maps for* C. albicans* and zebrafish would still benefit the construction of PPI networks and the investigation of essential functional modules in* C. albicans* infection in the future. Second, gene expression profiles were overlaid to estimate the expression of their corresponding proteins. However, there are several steps involved in the synthesis of proteins from mRNAs [74, 75]. The overlay of protein expression levels using gene expression values without any modification may result in inaccuracies in the identified PPI networks. Once high-throughput protein expression data are available, a great improvement in PPI network construction will be made. Third, the interactions of* C. albicans* with epithelial cells during the infection process can be roughly divided into three major steps: adhesion, invasion, and damage [76, 77]. Conversely, the host employs corresponding defensive mechanisms in response to invasive fungal infections, perhaps beginning with recognition, followed by defensive responses, and eventually a victor emerges from the competition of infection. However, due to limitations in the number of time points in the microarray data, the infection process was divided into only early and late stages in this study. If more time points in the microarray data could be obtained during the infection process, especially targeting the adhesion, invasion, and damage stages, more detailed stage-specific host-pathogen interactions could be investigated. In this way, the invasive and defensive strategies taken by* C. albicans* and zebrafish, respectively, could be more specifically elucidated.

Humans have to face a large number of challenges presented by pathogens during the course of a lifetime. Therefore, the investigation of molecular mechanisms of life-threatening infection is essential. For human fungal pathogens, the relevant research into infection could provide knowledge about network structures, infectious mechanisms, and bioecology [78]. Through a deeper understanding of pathogens, new therapeutic strategies against invasive microorganisms are also possible. However, few studies have explored the systems biology of pathogen infection and the host responses simultaneously. Hence, a comprehensive picture combining the pathogenic mechanisms of the pathogen and defensive mechanisms of its host could provide a novel antifungal drug discovery strategy to treat and prevent serious infectious disease, even mortality. In terms of the pathogen, the result of the analysis of* C. albicans* pathogenesis in this study highlights some functional modules associated with hyphal morphogenesis, ion and small molecule transport, protein secretion, and shifts in carbon utilization, in which all play crucial roles in invasion and damage to host cells. The molecules involved in these processes might be considered as potential targets for drug discovery. For hosts, the immune response, apoptosis, ion transport, protein secretion, and hemostasis-related processes were considered to be essential molecular mechanisms for defense and survival in* C. albicans* infections. Therefore, proteins involved in these functional modules could be therapeutically protected to prevent the irreparable damage caused by the infection. Most recently, we developed a computational framework to construct interspecies PPI network [79], which can be integrated with the current methodology to investigate the interspecies functional modules in the future. It is hoped, with the help of more detailed biological functional modules, that the treatment of life-threatening infection can be developed further and the mortality rates due to infection can ultimately be decreased.

## 5. Conclusions

In this study, with the help of simultaneous host-pathogen interaction microarrays for both* C. albicans* and zebrafish, we investigated essential functional modules for pathogenic and defensive mechanisms in* C. albicans* infections using differential network analysis. The early- and late-stage protein interaction networks for both organisms were first constructed. We then determined the network reconfiguration to identify the proteins with significant interaction variations during infection and to extract the enriched functional modules among these proteins. The hyphal morphogenesis, ion and small molecule transport, protein secretion, and shifts in carbon utilization functional modules in* C. albicans* were seen to play crucial roles in pathogen invasion and damage caused to host cells. The zebrafish functional modules like those involved in immune response, apoptosis mechanism, ion transport, protein secretion, and hemostasis-related processes were found to be significant as defensive mechanisms during* C. albicans* infection. The essential functional modules thus determined could provide insights into the molecular mechanisms during the infection process and thereby help to devise potential therapeutic strategies to treat* C. albicans *infection.

## Supplementary Material

The supplementary materials include supplementary methods and three supplementary figures. In the supplementary methods, the details of protein interaction network construction are provided. In the supplementary figures, the differential PPI networks and the distributions of structure variation values for both *C. albicans* and zebrafish are illustrated, respectively.

## Figures and Tables

**Table 1:** Enriched functional modules for pathogenic mechanisms in *C. albicans* and the corresponding significant proteins shown in Figure 2 during the host-pathogen interaction.

Functional module	Protein symbol
Hyphal morphogenesis	Cas4, Als3, Ifd6, Kis1, Mac4, Ndt80, orf19.6705

Ion and small molecule transport	Tna1, Agp2, Can1, Mac1, Als3, orf19.3769, Git1, Hut1, Mcd4, Mep1, Mrs7, orf19.1403, orf19.1427, orf19.2322.3, orf19.3132, orf19.3558, orf19.4897, Seo1, Sfc1, Vcx1

Protein secretion	Sap4, Sap5, Sap6, Sro77, Sys3, Ddi1, orf19.3247, orf19.7261, orf19.7604, orf19.841, Plb2, Prd1, Sec20, Spc3

Shifts in carbon utilization	Lat1, orf19.3782, Tes15, orf19.4121, Tes1, Pyc2, Acc1, Agp2, Pox1-3

This table lists four of the functional modules considered to be significant in *C. albicans *pathogenesis and the corresponding proteins with associated GO annotation.

**Table 2:** Enriched functional modules for defensive mechanisms in zebrafish and the corresponding significant proteins shown in Figure 4 during the host-pathogen interaction.

Functional module	Protein symbol
Immune response	Tlr2, B2m, Akt2, Akt2l, Apaf1, Cxcr3.2, Pik3r3a, Sigirr, Ticam1, Tlr20a, Vtna

Apoptosis mechanism	Akt2, Akt2l, Apaf1, Cdk5, Gdnfa, Nras, Phlda3, Pik3r3a, Plcg2, Prkar2ab, Sgk1, Tax1bp1a

Ion transport	Tfa, Abcc9, Cacnb3a, Cacnb4b, Clk2a, Cox5ab, Grid2, Grin1a, Grin1b, Kcnh1, Kcnq1, Sfxn1, si:ch211-12e13.7, si:ch211-258f14.5, Slc12a3, Slc26a6l, Slc39a6, Trpc6a, Trpm7, zgc:109934, zgc:162160

Protein secretion	Rab2a, Rab3da, Rab3db, Rab6ba, Rab8a, Rab10, Rab11a, Rab35, Ap1m2, Ap3m1, Atg4c, Bcap31, Naca, Nup85, Ramp2, Scamp2, Snx17, Tnpo2, Trpc4apa, zgc:113338

Hemostasis-related processes	Calcrla, Lama4, Acvrl1, Bmp4, Cdh2, Csrp1a, Ell, Gata2a, Hapln1b, Hopx, Nr2f2, Nrxn3a, Nrxn3b, Plxnb2a, Rab11a

The table lists five of the functional modules considered to be essential in the *C. albicans*-zebrafish interaction and the corresponding proteins with associated GO annotation.
