# Supplementary material for: Essential Functional Modules for Pathogenic and Defensive Mechanisms in Candida albicans Infections
Source: Biomed Res Int. 2014 Mar 18;2014:136130. doi: 10.1155/2014/136130 (PMC3976935; doi:10.1155/2014/136130)

# Supplementary Materials

# Essential Functional Modules for Pathogenic and Defensive Mechanisms in *Candida albicans* Infections

## Supplementary methods

### Details of protein interaction network construction

A discrete dynamic model was employed to determine the PPI networks that occurred in the infection of zebrafish by *C. albicans*. For a target protein *i* in the rough PPI network, the dynamic model of the activity of protein *i* was calculated as follows [1]:

$$z_i[t+1] = z_i[t] + \sum_{j=1}^{M_i} b_{ij} z_i[t] z_j[t] + \alpha_i x_i[t] - \beta_i z_i[t] + h_i + \omega_i[t] \quad \text{(S1)}$$

where $z_i[t]$ represents the protein expression level of the target protein *i* at time *t*, $b_{ij}$ denotes the ability of the *j*-th interactive protein to interact with target protein *i*, $z_j[t]$ indicates the expression level of protein *j* which interacts with the target protein *i* at time *t*, $\alpha_i$ represents the translation effect of mRNA to protein, $x_i[t]$ is the corresponding mRNA expression level of the target protein *i*, $\beta_i$ denotes the degradation rate of *i*, $h_i$ indicates the basal level of *i*, and $\omega_i[t]$ represents the stochastic noise. The biological significance of equation S1 is that the protein expression level of target protein *i* at a later time *t*+1 is a function of the protein expression level occurring at time *t*, the regulatory interactions with $M_i$ interactive proteins, the process of translation from mRNA, the effects of protein degradation, the basal level of protein *i*, and the stochastic noises [1]. To identify the associated parameters using microarray data, a constrained least squares parameter estimation was adopted [1]. Equation S1 can be represented in the following regression form:

$$z_i[t+1] = \begin{bmatrix} z_i[t]z_1[t] & \cdots & z_i[t]z_{M_i}[t] & x_i[t] & z_i[t] & 1 \end{bmatrix} \begin{bmatrix} b_{i1} \\ \vdots \\ b_{iM_i} \\ \alpha_i \\ 1-\beta_i \\ h_i \end{bmatrix} + \omega_i[t] \quad \text{(S2)}$$

$$= \phi_i[t] \cdot \theta_i + \omega_i[t]$$

where $\phi_i[t]$ represents the regression data vector and $\theta_i$ denotes the kinetic parameter vector to be estimated. In order to avoid the danger of overfitting the estimated parameters, the original

data points were interpolated to $L$ data points by the cubic spline method. In other words, there were $\{z_i[l+1],\ \phi_i[l]\}$ data point pairs for $l \in \{1, \ \ldots \ , L-1\}$. Hence equation S2 can be written in the following form for target protein $i$:

$$Z_i = \Phi_i \cdot \theta_i + \Omega_i \tag{S3}$$

where $Z_i = \begin{bmatrix} z_i[2] \\ \vdots \\ z_i[L] \end{bmatrix}$, $\Phi_i = \begin{bmatrix} \phi_i[1] \\ \vdots \\ \phi_i[L-1] \end{bmatrix}$, $\Omega_i = \begin{bmatrix} \omega_i[1] \\ \vdots \\ \omega_i[L-1] \end{bmatrix}$.

In this case the parameter estimation problem for target protein $i$ in the rough PPI network can be represented by the following constrained least squares minimization equation:

$$\min_{\theta_i} \frac{1}{2} \left\| \Phi_i \cdot \theta_i - Z_i \right\|_2^2 \text{ such that } C \cdot \theta_i \le d \tag{S4}$$

where $C = \mathrm{diag}[0 \ \cdots \ 0 \ -1 \ 0 \ -1]$ and $d = [0 \ \cdots \ 0]^T$, constraining the parameters $\alpha_i$ and $h_i$ to be non-negative.

Once the interaction abilities $b_{ij}$ were estimated protein by protein in the rough PPI network using the constrained least squares parameter estimation method, the Akaike Information Criterion (AIC) [2, 3] was applied to prune those insignificant interactions in the rough PPI network by the system order detection technique. AIC, which includes both estimated residual error and model complexity in one statistics, quantifies the relative goodness of fit of a model. For a protein interaction model with $M_i$ regulatory interaction parameters (or proteins) to fit with data from $L$ samples, the AIC can be written as follows [2, 3].

$$\mathrm{AIC}(M_i) = \log\left( \frac{1}{L} \left( Z_i - \hat{Z}_i \right)^T \left( Z_i - \hat{Z}_i \right) \right) + \frac{2M_i}{L} \tag{S5}$$

where $\hat{Z}_i$ denotes the estimated expression profile of the $i$-th target protein, i.e. $\hat{Z}_i = \Phi_i \cdot \hat{\theta}_i$, and $\hat{\sigma}_i^2 = \frac{1}{L} \left( Z_i - \hat{Z}_i \right)^T \left( Z_i - \hat{Z}_i \right)$ is the estimated residual error. As the residual error $\hat{\sigma}_i^2$ decreases, the AIC decreases. In contrast, while the number of interactive proteins (or parameters) $M_i$ increases, the AIC increases. Therefore, there is a tradeoff between residual error and model order. As the expected residual error decreases with increasing interactive protein numbers in models of inadequate complexity, there should be a minimum around the optimal interactive protein number. The minimization achieved in equation S5 will indicate the ideal model order (i.e. the optimal number of proteins that interact with the target protein) of the protein interaction system. With the statistical selection of $M_i$ interactive proteins by minimization of the AIC, the question of whether an interactive protein is a significant one or

just a false positive for the *i*-th target protein can be determined. Hence, AIC can be adopted to select model order, filtering out insignificant protein interactions in the rough PPI network producing a more refined PPI network based on the estimated interaction abilities ($b_{ij}$s) obtained by time profile microarray data. In this way, with different sets of microarray data (0.5~4 hpi for the early stage and 4~18 hpi for the late stage), two refined PPI networks were constructed for the early and late stages of *C. albicans* infection of zebrafish by removing insignificant interactions through AIC for both organisms.

## References


[1] Y. C. Wang and B. S. Chen, "Integrated cellular network of transcription regulations and protein-protein interactions," *BMC Syst Biol*, vol. 4, pp. 20, 2010.

[2] R. Johansson, *System modeling and identification*, Prentice Hall, Englewood Cliffs, NJ, 1993.

[3] H. Akaike, "New look at statistical-model Identification," *IEEE Transactions on Automatic Control*, vol. 19, no. 6, pp. 716-723, 1974.

# Supplementary figure S1

The differential PPI network obtained from the early and late stage PPI networks of *C. albicans*. Red and blue edges indicate positive and negative $d_{ij,l}$ values respectively, calculated using equation 2. The protein names have been omitted for simplicity.

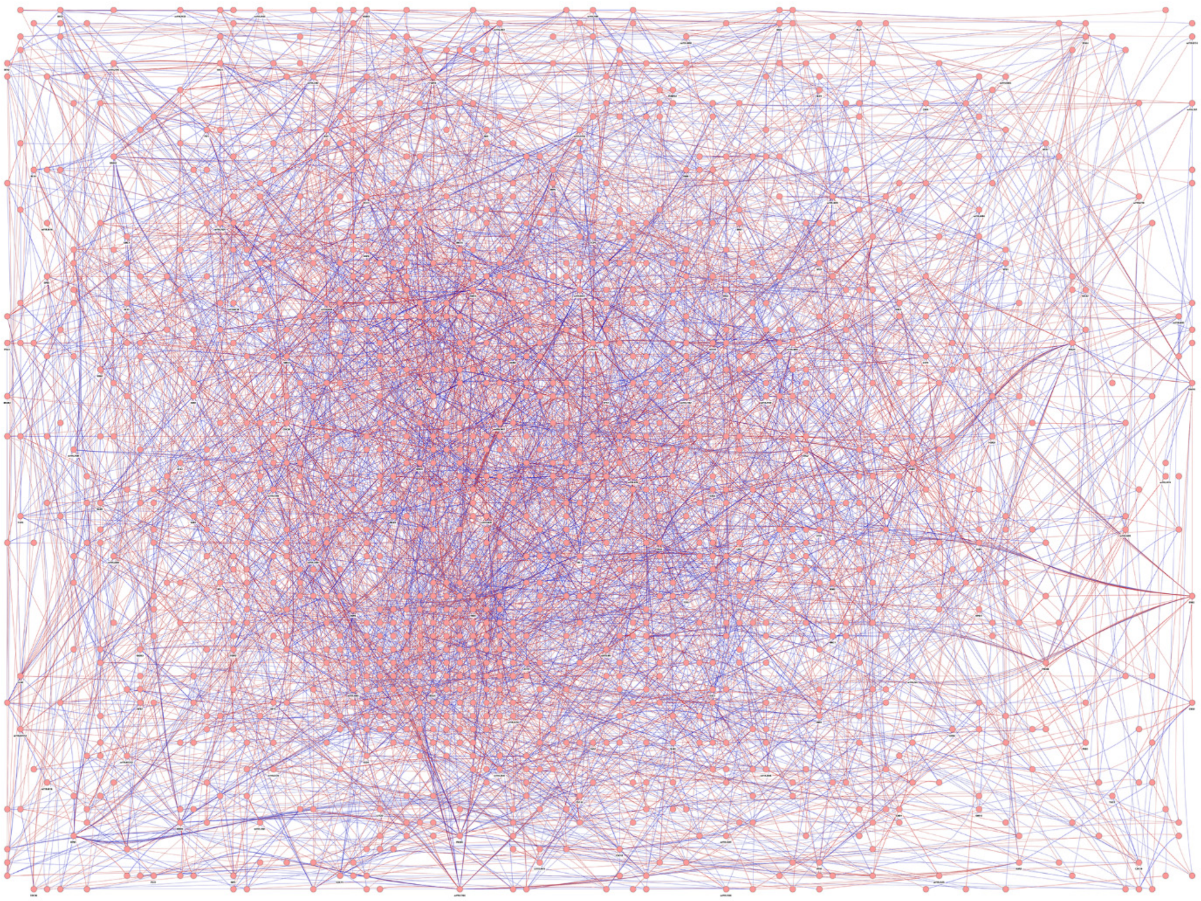

## Supplementary figure S2

The differential PPI network obtained from the early and late stage PPI networks of zebrafish. Red and blue edges indicate positive and negative $d_{ij,l}$ values respectively, calculated using equation 2. The protein names have been omitted for simplicity.

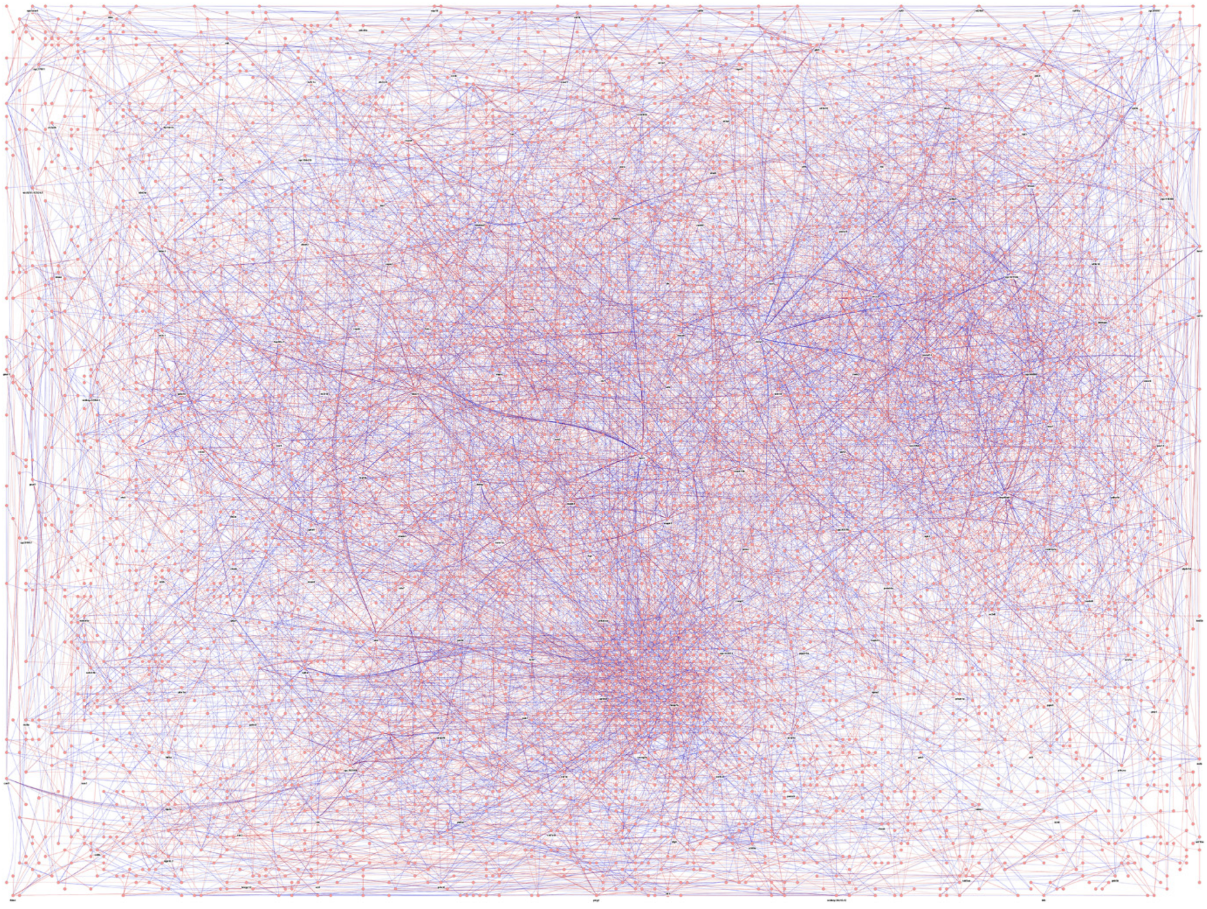

## Supplementary figure S3

Distributions of structure variation values (SVVs) for *C. albicans* and zebrafish.

(A) *C. albicans* (B) zebrafish

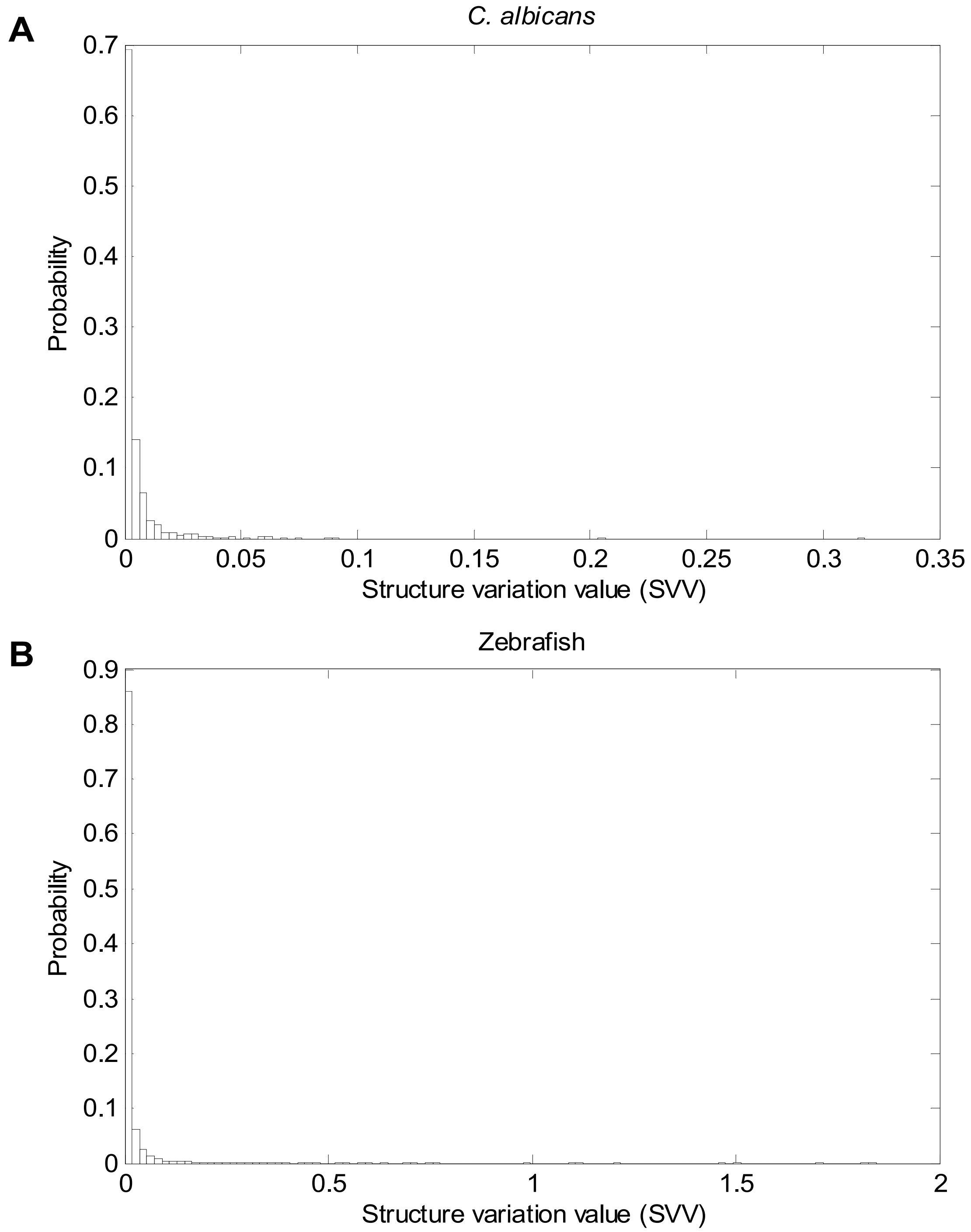

Supplement: Supplementary file 1 — The supplementary materials include supplementary methods and three supplementary figures. In the supplementary methods, the details of protein interaction network construction are provided. In the supplementary figures, the differential PPI networks and the distributions of structure variation values for both C. albicans and zebrafish are illustrated, respectively. [file 136130.f1.pdf]
